# Specific Expression of Human Intelectin-1 in Malignant Pleural Mesothelioma and Gastrointestinal Goblet Cells

**DOI:** 10.1371/journal.pone.0039889

**Published:** 2012-07-02

**Authors:** Kota Washimi, Tomoyuki Yokose, Makiko Yamashita, Taihei Kageyama, Katsuo Suzuki, Mitsuyo Yoshihara, Yohei Miyagi, Hiroyuki Hayashi, Shoutaro Tsuji

**Affiliations:** 1 Department of Pathology, Kanagawa Cancer Center, Yokohama, Japan; 2 Molecular Diagnostic Project, Kanagawa Cancer Center Research Institute, Yokohama, Japan; 3 Division of Molecular Pathology and Genetics, Kanagawa Cancer Center Research Institute, Yokohama, Japan; 4 Department of Pathology, Yokohama Municipal Citizen’s Hospital, Yokohama, Japan; The Chinese University of Hong Kong, Hong Kong

## Abstract

Malignant pleural mesothelioma (MPM) is a fatal tumor. It is often hard to discriminate MPM from metastatic tumors of other types because currently, there are no reliable immunopathological markers for MPM. MPM is differentially diagnosed by some immunohistochemical tests on pathology specimens. In the present study, we investigated the expression of intelectin-1, a new mesothelioma marker, in normal tissues in the whole body and in many cancers, including MPM, by immunohistochemical analysis. We found that in normal tissues, human intelectin-1 was mainly secreted from gastrointestinal goblet cells along with mucus into the intestinal lumen, and it was also expressed, to a lesser extent, in mesothelial cells and urinary epithelial cells. Eighty-eight percent of epithelioid-type MPMs expressed intelectin-1, whereas sarcomatoid-type MPMs, biphasic MPMs, and poorly differentiated MPMs were rarely positive for intelectin-1. Intelectin-1 was not expressed in other cancers, except in mucus-producing adenocarcinoma. These results suggest that intelectin-1 is a better marker for epithelioid-type MPM than other mesothelioma markers because of its specificity and the simplicity of pathological assessment. Pleural intelectin-1 could be a useful diagnostic marker for MPM with applications in histopathological identification of MPM.

## Introduction

Malignant pleural mesothelioma (MPM) is a fatal tumor. The median survival of patients with MPM after chemotherapy or radical surgery is only 9–12 months [1]. Since MPM is correlated with prior exposure to asbestos [2], the number of people with MPM is increasing relative to the amount of asbestos used in the world [3], [4]. It is often difficult to diagnose MPM and to distinguish it from metastatic carcinomas because a sensitive and reliable diagnostic marker for MPM has not been found yet. The differential diagnosis of MPM depends on some immunohistochemical tests of surgical pathology specimens. Calretinin, cytokeratin 5/6 (CK5/6), mesothelin, Wilm’s tumor gene product 1 (WT-1), and podoplanin, often referred to as D2-40, have been reported as immunohistochemical markers for epithelioid-type MPM [5]–[7]. Recently, we reported that a large amount of intelectin-1 is secreted into the malignant pleural fluid from epithelioid-type MPMs [8].

The intelectin gene was shown to be specifically expressed in cells of the small intestine [9]. Intelectin binds to galactofuranosyl residues and pathogens [10], [11], and infection or inflammation causes an increase in the expression of intelectin mRNA in the intestine [12], [13] or bronchus [14]. Human intelectin-1 is expressed in intestinal goblet cells, besides in MPMs, and is present at about 200 ng/mL in serum [8]. Intelectin-1 was also shown to be expressed in human endothelial cells [15] and adipocytes of human abdominal adipose tissue [16]. However, intelectin-1 expression in normal or cancerous tissues in humans has not been investigated yet with human intelectin-1-specific antibodies.

In the present study, we analyzed intelectin-1 expression in human tissues lacking evident pathological lesions, and in some cancerous tissues, by immunohistochemical analysis using a monoclonal antibody against human intelectin-1. Intelectin-1 was expressed in gastrointestinal goblet cells, kidney collecting tubule cells, some bladder umbrella cells, and some mesothelial cells. Almost all epithelial-type MPMs (23/26) expressed intelectin-1, as well as other MPM markers. On the other hand, sarcomatoid-type MPMs, biphasic MPMs, and poorly differentiated MPMs rarely stained positive with anti-intelectin-1. Intelectin-1 was not expressed in the other cancers, except in some mucus-producing adenocarcinomas. Mucus-producing metastatic cancer is rare in the pleura, and it is easy to discriminate between mucus-producing adenocarcinomas and MPMs in the pleura, histopathologically. Thus, these results suggested that pleural intelectin-1 could be a useful diagnostic marker in the histopathological evaluation of MPM.

## Materials and Methods

All experimental protocols were approved by the ethics committees of the Kanagawa Cancer Center. Written informed consent for this study was obtained from each patient. The written consent for the patient anatomized after death was obtained from the next of kin or guardian of the patient. All data were analyzed anonymously throughout the study.

### Tissues

We obtained tissue samples of the cholecyst and breast from patients surgically treated at the Kanagawa Cancer Center between 2006 and 2010. Other non-tumorous tissue samples were obtained from patient specimens that were anatomized within 4 h after death at the Kanagawa Cancer Center between 2006 and 2010. Mesothelioma samples were obtained from patients who were diagnosed with malignant mesothelioma at the Kanagawa Cancer Center or Yokohama Municipal Citizen’s Hospital between 1998 and 2011. Other cancer tissues were obtained from the primary tumors of the patients who were pathologically diagnosed as having cancer at the Kanagawa Cancer Center between 1999 and 2012. The pathology specimens used included the following cancers: biphasic synovial sarcoma, breast cancer, colon adenocarcinoma, epithelioid angiosarcoma, epithelioid hemangioendothelioma, gastric adenocarcinoma, lung cancer, ovarian adenocarcinoma, renal cell carcinoma, and urothelial carcinoma.

Normal tissues, which did not have evident pathological lesions, were extracted from organs at an average time of 8 d after formalin fixation. The cancer tissues were prepared from formalin-fixed samples. All specimens were prepared as paraffin-embedded thin-sliced sections. The specimens, except the mesotheliomas, were used as tissue microarrays, with the tissue samples cut to a circle 1 or 3 mm in diameter.

### Immunohistochemistry

The sections on glass slides were deparaffinized and rehydrated with xylene and ethanol and then heated at 95°C for 40 min (intelectin-1, calretinin, CK5/6, mesothelin, podoplanin, and S-100 protein) or 121°C for 10 min (WT-1) in an antigen-retrieval buffer (10 mM Tris buffer [pH 9.0] containing 1 mM ethylenediaminetetraacetic acid [intelectin-1, CK5/6, podoplanin, S-100 protein, and WT-1], or 10 mM citrate buffer [pH 6.0] containing 0.1% Tween-20 [calretinin and mesothelin]). Endogenous peroxidase activity was eliminated by incubating the sections with 3% hydrogen peroxide for 5 min. After washing the sections with phosphate-buffered saline, the sections were treated with primary antibodies for 1 h. They were then washed with 20 mM Tris-buffered saline (pH 7.6) containing 0.05% Tween 20, and immunoreactivity was visualized by using Histofine Simple Stain MAX-PO (Multi) (Nichirei Co., Tokyo, Japan) with the Liquid DAB+ (Dako Japan Co., Ltd., Kyoto, Japan) or the EnVision+ kits (Dako Japan Co., Ltd.) according to the manufacturer’s instructions. The sections were finally counterstained with hematoxylin, dehydrated, and mounted with Malinol medium (Muto Pure Chemicals Co., Ltd., Tokyo, Japan). The primary antibodies used were as follows: intelectin-1, mouse anti-human intelectin-1 monoclonal antibody (3G9) (Immuno-Biological Laboratories Co., Ltd., Gunma, Japan); calretinin, rabbit anti-calretinin polyclonal antibody (PAD:DC8) (Life Technologies Japan Ltd., Tokyo, Japan); CK5/6, mouse anti-cytokeratin 5,6 monoclonal antibody (D5/16B4) (Dako Japan Co., Ltd.); mesothelin, mouse anti-human mesothelin monoclonal antibody (5B2) (Leica Microsystems, Inc., Bannockburn, IL); podoplanin, mouse anti-podoplanin monoclonal antibody (D2-40) (Nichirei Co.); S-100 protein, mouse anti-S-100 protein monoclonal antibody (4C4.9) (Ventana Medical Systems, Inc., Tucson, AZ); WT-1, mouse anti-human WT-1 monoclonal antibody (6F-H2) (Dako Japan Co., Ltd.). Each antibody was used according to the respective manufacturer’s instructions.

Immunostaining was evaluated on the basis of the intensity and proportion of staining on all cancer cells in each specimen. For evaluating the immunostaining for calretinin or WT-1, the staining in the nucleus, but not the cytoplasm, was measured. The intensity of staining was defined by applying the Allred scoring [17] as follows: 3+, strong staining; 2+, moderate staining; 1+, weak staining; –, no staining. The proportion of staining was measured in the entire microscopic field of cancer cells for each specimen and was classified by applying the Allred scoring as follows: 5, >66%; 4, 66–33%; 3, 33–10%; 2, 10–1%; 1, <1%; 0, 0%. Cases were defined as positive if the Allred score of the marker, which is the value of the intensity score plus the proportion score, was more than 3.

## Results

### Intelectin-1 Expression in Normal Tissues

**Figure 1:**
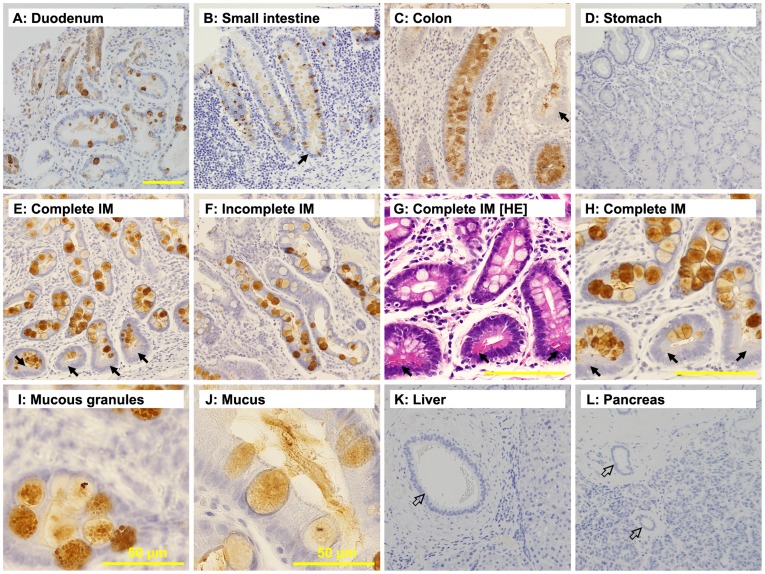
Expression of intelectin-1 in gastrointestinal goblet cells. Specimens were immunostained with anti-intelectin-1, and representative photographs are shown here. The scale bar (100 µm) is shown at the bottom of panel A, representatively. The scale bars of panel G (100 µm), panel H (100 µm), panel I (50 µm), and panel J (50 µm) are also shown. The arrows indicate paneth cells. **A**, duodenum; **B**, small intestine; **C**, colon; **D**, stomach; **E**, complete intestinal metaplasia (IM) in stomach; **F**, incomplete IM in stomach; **G**, hematoxylin–eosin (HE) staining of complete IM in stomach; **H**, complete IM in stomach; **I**, mucous granules in goblet cells; **J**, mucus-secreting goblet cells; **K**, liver and bile duct (open arrow); **L**, pancreas and pancreatic duct (open arrows).

Intelectin-1 expression in normal tissues was investigated by immunohistochemical analysis using an anti-intelectin-1 monoclonal antibody. The results are summarized in Table 1. Intelectin-1 was produced by goblet cells in the duodenum (Figure 1A), the small intestine (Figure 1B), and the colon (Figure 1C), but not normal gastric tissue (Figure 1D). Intelectin-1 was also expressed in goblet cells in intestinal metaplasia in the stomach. The goblet cells produced intelectin-1 in complete and incomplete intestinal metaplasias (Figure 1E and 1F). Paneth cells in the small intestine (Figure 1B, arrow), the colon (Figure 1C, arrow), and complete intestinal metaplasia (Figure 1G and 1H, arrows) did not express intelectin-1. Intelectin-1 was pooled in mucous granules in goblet cells (Figure 1I) and was secreted with mucus into the intestinal lumen (Figure 1J). The mucus was not stained with negative control antibody (Figure S1). Bile duct in the liver (Figure 1K, open arrow), pancreatic duct in the pancreas (Figure 1L, open arrows), and other tested components of the digestive system did not express intelectin-1 (Table 1).

**Table 1:** Intelectin-1 expression in normal tissues.

Organ		Intensity of intelectin-1 staining(No. of score/No. of samples)				Positive cell
		3+	2+	1+	–	
Digestive system	Tongue				1/1	
	Esophagus				2/2	
	Stomach (normal tissue)				3/3	
	Stomach (IM)	9/9				Goblet cells
	Duodenum	2/2				Goblet cells
	Small intestine	4/4				Goblet cells
	Colon	9/9				Goblet cells
	Appendix				2/2	
	Liver				8/8	
	Pancreas				11/11	
	Cholecyst				6/6	
Urinary system	Bladder		1/4		3/4	Umbrella cells
	Kidney			6/10	4/10	Collecting tubule cells
	Ureter				1/1	
Mesothelium	Pleura		3/10		7/10	Mesothelial cells
	Peritoneum		1/2		1/2	Mesothelial cells
	Tunica vaginalis		1/2		1/2	Mesothelial cells
	Pericardium		1/1			Mesothelial cells
Endocrine organ	Pituitary gland				5/5	
	Adrenal			3/5	2/5	Cortex cells
	Thyroid				9/9	
	Parathyroid				3/3	
	Adipose tissue*				5/5	
	Greater omentum		1/2		1/2	Mesothelial cells
Vascular	Aorta				3/3	
	Vascular endothelium*				5/5	
Nervous system	White substance				13/13	
	Gray substance				16/16	
	Ventricle				4/4	
	Basal nucleus				2/2	
	Nigra				2/2	
	Cerebellum				1/1	
	Spinal cord				1/1	
	Dura mater				1/1	
	Nerve plexus				1/1	
Respiratory system	Bronchus			5/15	10/15	Bronchial mucous gland
	Lung				36/36	
	Diaphragm				2/2	
Lymphoid system	Lymph node				6/6	
	Thymus gland				3/3	
	Spleen				3/3	
	Bone marrow				7/7	
Reproductive system	Breast				6/6	
	Ovary				4/4	
	Uterus				4/4	
	Vaginal				1/1	
	Testis				6/6	
	Prostate				11/11	
Muscle tissue	Skeletal muscle				1/1	
	Smooth muscle				3/3	
	Cardiac muscle				19/19	
Skin	Epidermis				1/1	
Bone	Spondylus				7/7	

Expression scores were assigned by evaluating the intensity of intelectin-1 staining on the positive cells. The intensity of staining was defined by applying the Allred scoring as follows: 3+, strong staining; 2+, moderate staining; 1+, weak staining; –, no staining. The asterisk indicates the tissue in the digestive, urinary, respiratory, or reproductive system. IM, intestinal metaplasia.

**Figure 2:**
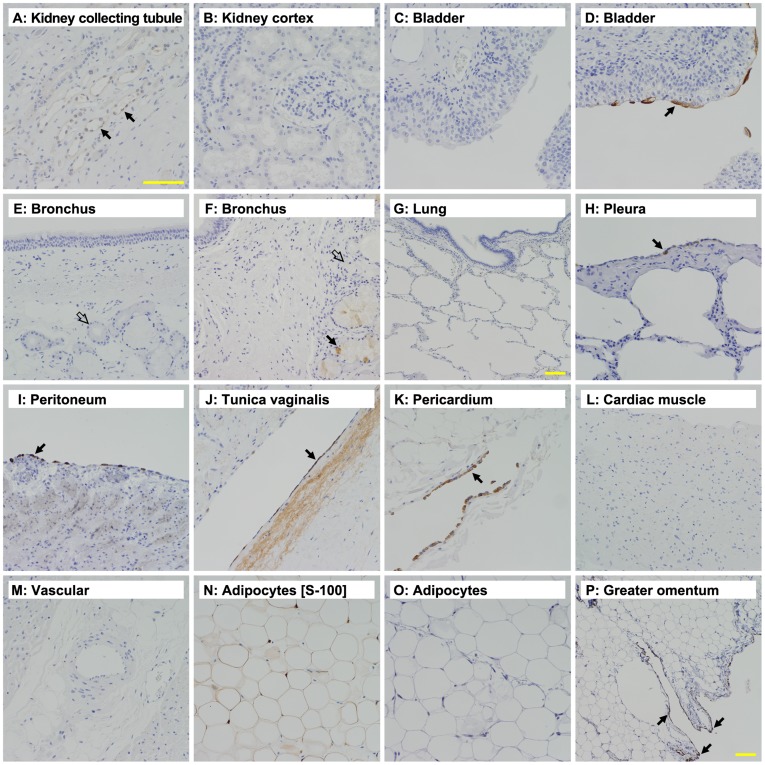
Expression of intelectin-1 in normal tissues. Specimens were immunostained with anti-intelectin-1, and representative photographs are shown. A scale bar (100 µm) is shown in panel A, representatively. The scale bars of panel G (100 µm) and panel P (100 µm) are also shown. The closed black arrows indicate intelectin-1-positive cells. **A**, kidney collecting tubule; **B**, kidney cortex; **C**, intelectin-1-negative bladder; **D**, intelectin-1-positive bladder; **E**, bronchus. The open arrow shows the serous gland; **F**, bronchus. The open or closed arrow shows the serous or mucous gland, respectively; **G**, lung; **H**, intelectin-1-positive pleura; **I**, intelectin-1-positive peritoneum; **J**, intelectin-1-positive tunica vaginalis; **K**, pericardium; **L**, cardiac muscle and endocardium; **M**, vascular; **N**, S-100 protein staining of adipocytes in greater omentum; **O**, adipocytes in greater omentum; **P**, greater omentum with mesothelial cells.

In the urinary system, intelectin-1 was weakly expressed in the collecting tubule cells in the kidney (Figure 2A, arrow) but was detected neither in the glomerulus nor in other kidney cells (Figure 2B). Although almost all cells in the bladder were intelectin-1-negative (Figure 2C), a donor exhibited intelectin-1 expression in umbrella cells in the bladder (Figure 2D, arrow). Bronchial epithelium including goblet cells (Figure 2E), serous glands (Figure 2E and 2F, open arrows), and lung (Figure 2G) did not express intelectin-1. On the other hand, mucous cells in the bronchial mucous gland (Figure 2F, closed arrow) and mesothelial cells in the pleura (Figure 2H), peritoneum (Figure 2I), tunica vaginalis (Figure 2J), or pericardium (Figure 2K) often expressed intelectin-1. Intelectin-1 expression was not observed in cardiac muscle, endocardium (Figure 2L), or vascular endothelial cells (Figure 2M). S-100 protein, which is expressed in adipocytes [18], was detected in the cytoplasm of adipocytes in abdominal adipose tissue (Figure 2N), whereas intelectin-1 was not detected in adipocytes (Figure 2O). Intelectin-1 expression was observed in the mesothelial cells on adipose tissue rather than in adipocytes (Figure 2P, arrow). Adrenal cortex was weakly stained with anti-intelectin-1 (Table 1). Intelectin-1 was not expressed in other tested tissues, including endocrine organs, muscle tissue, skin, bone, and the nervous, respiratory, lymphoid, and reproductive systems (Table 1).

These results suggest that intelectin-1 is mainly secreted from intestinal goblet cells along with mucus into the intestinal lumen and that mesothelial cells and epithelial cells in the urinary system produce intelectin-1 only occasionally, in response to some stimulatory signals.

### Intelectin-1 Expression in Mesotheliomas

**Figure 3:**
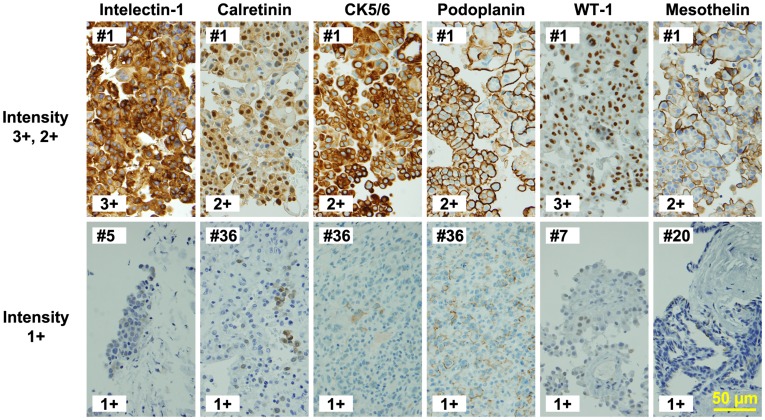
Immunostaining of mesothelioma markers in MPM. A scale bar (50 µm) is shown at the bottom right. The patient ID (Table S1) is shown at the upper left on each panel.

Epithelioid-type MPMs were immunostained with antibodies against intelectin-1 or typical mesothelioma markers, such as calretinin, CK5/6, podoplanin, WT-1, and mesothelin. Representative images of immunostaining against these antigens are shown in Figure 3. The results are summarized in Table 2, and the individual data are shown in Table S1. Eighty-eight percent (23/26 cases) of epithelioid-type MPMs were stained positively with anti-intelectin-1 (Table 2). As shown in Figure 3, intelectin-1 and CK5/6 were detected in the cytoplasm. Both the cytoplasm and the nucleus stained positive for calretinin and WT-1, but the staining in the nucleus was important for distinction of MPM cells. Podoplanin and mesothelin were mainly present on the cell membrane. The average Allred score of intelectin-1 (5.8) in epithelioid-type MPM was similar to that of calretinin (6.0), CK5/6 (6.2), podoplanin (5.5), WT-1 (5.9), and mesothelin (5.7) (Table 2). The proportion of intelectin-1-positive cells in epithelioid-type MPMs was higher than that of other markers (Table S1). Sarcomatoid-type MPMs, poorly differentiated MPMs, or biphasic MPMs were rarely stained with anti-intelectin-1 (Table 2). These results suggest that intelectin-1 is a sensitive marker for histopathological detection of epithelioid-type MPMs.

**Table 2:** Expression of marker antigens in MPM.

MPM type			Intelectin-1	Calretinin	CK5/6	Podoplanin	WT-1	Mesothelin
Epithelioid			23/26	26/26	24/26	23/26	23/26	25/26
	Allred score	0	2	0	1	2	2	0
		2	0	0	0	0	0	0
		3	1	0	1	1	1	1
		4	3	3	2	1	0	6
		5	2	6	2	5	3	4
		6	3	5	3	8	5	6
		7	11	11	14	9	14	8
		8	4	1	3	0	1	1
		Average	5.8	6.0	6.2	5.5	5.9	5.7
PD			1/3	1/3	3/3	2/3	2/3	2/3
Biphasic			1/3	3/3	3/3	2/3	2/3	1/3
Sarcomatoid			0/7	0/7	2/7	4/7	5/7	0/7

The histologic type of MPM was classified by pathological diagnosis. The intensity and proportion of staining of MPM cells were evaluated in the entire microscopic field of the specimen. The individual data are described in Table S1. Cases were defined as positive if the Allred score, which is the value of the intensity score plus the proportion score, of the marker was more than 3. In the immunostaining of calretinin or WT-1, staining in the nucleus, but not the cytoplasm, was designated as a positive sample. PD, poorly differentiated.

### Insignificant Expression of Intelectin-1 in Other Cancers

**Figure 4:**
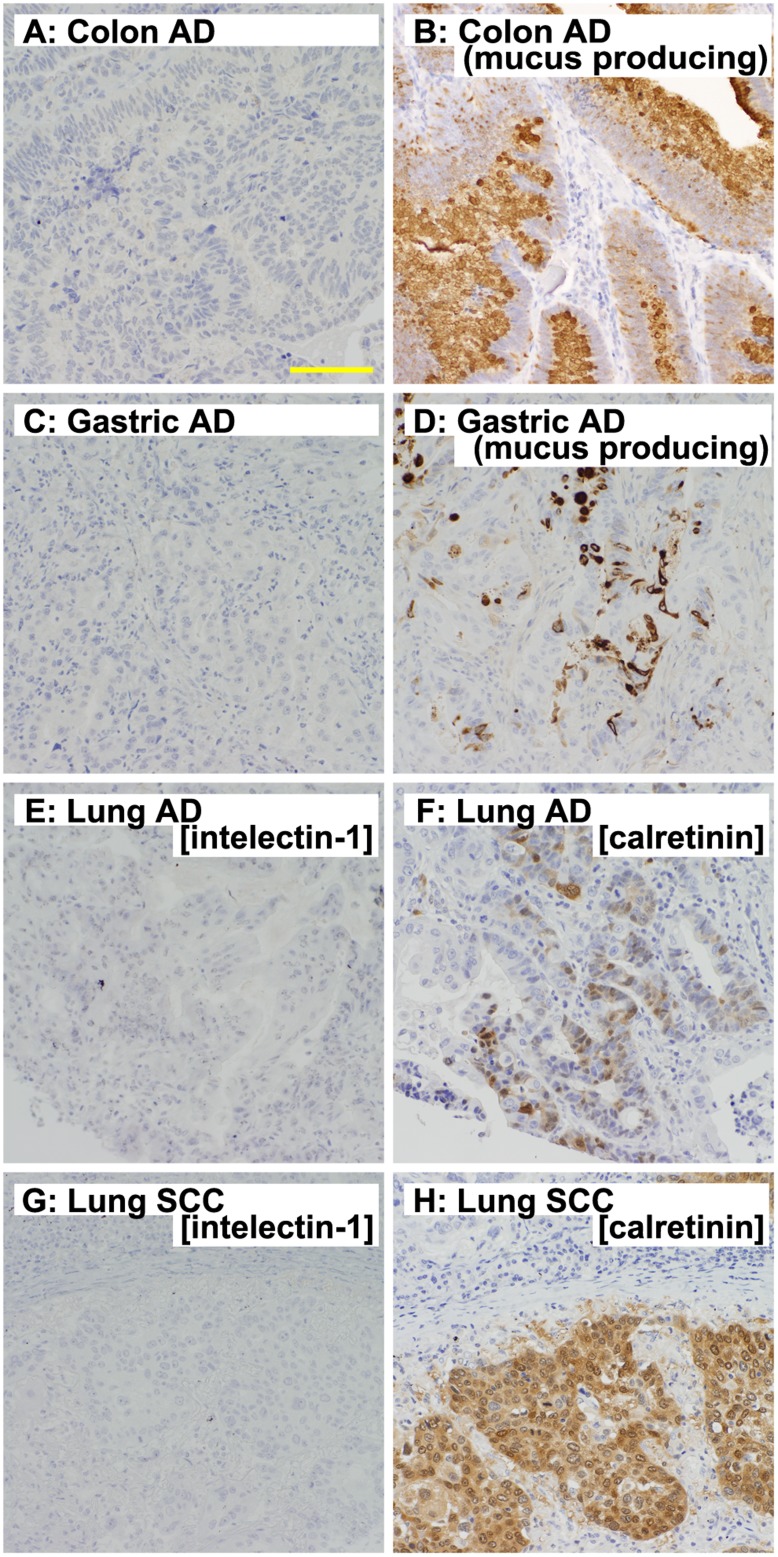
Insignificant expression of intelectin-1 in non-MPM cancers that lack mucus production. Specimens were immunostained with MPM markers, and representative photographs are shown. A scale bar (100 µm) is shown in the bottom side of panel A, representatively. **A**, intelectin-1 staining of a colon adenocarcinoma (AD) lacking mucus production; **B**, intelectin-1 staining of mucus-producing colon AD; **C**, intelectin-1 staining of a gastric AD lacking mucus production; **D**, intelectin-1 staining of mucus-producing gastric AD; **E**, intelectin-1 staining of lung AD; **F**, calretinin staining of lung AD; **G**, intelectin-1 staining of lung squamous cell carcinoma (SCC); **H**, calretinin staining of lung SCC.

To investigate intelectin-1 expression in cancers that may be confused with MPM in histopathological analyses, we prepared tissue microarrays of several primary tumors: biphasic synovial sarcoma, breast cancer, colon adenocarcinoma, epithelioid angiosarcoma, epithelioid hemangioendothelioma, gastric adenocarcinoma, lung cancer, ovarian adenocarcinoma, renal cell carcinoma, and urothelial carcinoma. As shown in Figure 4 and Table 3, intelectin-1 was not expressed significantly in these cancers, except for some mucus-producing adenocarcinomas (Figure 4B, 4D, and Table 3, marked with an asterisk). The differential diagnosis between lung cancer and MPM is often a serious problem. Intelectin-1 was expressed in only 1 of 88 cases of lung cancer, whereas calretinin, CK 5/6, podoplanin, and mesothelin were expressed in about 23%, 44%, 14%, and 40% of lung cancers, respectively (Table 3). Representative images of intelectin-1 and calretinin staining of lung cancer are shown in Figure 4E–H. Calretinin, CK 5/6, podoplanin, or mesothelin was often expressed in other cancers (Table 3). The nuclear staining for WT-1 was specifically observed in MPM (Table 3), but 64% of other cancers stained for WT-1 in the cytoplasm. Furthermore, the cytoplasm of normal cells was often strongly stained for WT-1 in many tissues (data not shown). Because of the cytoplasmic staining for WT-1, the nuclear staining for WT-1 could not be distinguished very clearly.

**Table 3:** Expression rate of mesothelioma markers in some cancers.

Cancer (n)	Histologic subtype	Intelectin-1	Calretinin	CK5/6	Podoplanin	WT-1	Mesothelin
Lung cancer (88)		1/88	20/88	39/88	12/88	0/88	35/88
	AD, acinar (40)	0	9	12	2	0	17
	AD, lepidic (2)	0	1	0	0	0	0
	AD, micropapillary (6)	0	0	2	1	0	4
	AD, papillary (10)	0	2	3	0	0	2
	AD, solid (10)	1	1	2	0	0	6
	SCC (20)	0	7	20	9	0	6
RCC (10)		0/10	0/10	1/10	0/10	0/10	0/10
UC (10)		1/10	2/10	7/10	0/10	0/10	0/10
Gastric AD (10)		4/10	2/10	7/10	6/10	0/10	1/10
	*Mucous (6)	4	0	5	5	0	1
	Non-mucous (4)	0	2	2	1	0	0
Colon AD (11)		*1/11	1/11	1/11	0/11	0/11	0/11
Breast cancer (10)		0/10	1/10	2/10	0/10	1/10	0/10
Ovarian AD (10)		*1/10	0/10	7/10	1/10	3/10	6/10
EHE (6)		0/6	0/6	3/6	2/6	0/6	0/6
EAS (2)		0/2	2/2	2/2	1/2	0/2	0/2
BSS (5)		0/5	4/5	4/5	2/5	0/5	2/5

Intensity and proportion of staining in the cancer cells were evaluated in the entire microscopic field of each specimen. Cases were defined as positive if the Allred score of the marker was more than 3. In the immunostaining of calretinin or WT-1, staining in the nucleus, but not the cytoplasm, was designated as a positive sample. Calretinin or WT-1 was detected in the cytoplasm of 14% or 64% of cancers, respectively. The asterisk marks the sample from a mucus-producing cancer. AD, adenocarcinoma; BSS, biphasic synovial sarcoma; EAS, epithelioid angiosarcoma; EHE, epithelioid hemangioendothelioma; RCC, renal cell carcinoma; SCC, squamous cell carcinoma; UC, urothelial carcinoma.

Histopathologically, it would be easy to distinguish the mucus-producing adenocarcinoma, which expressed intelectin-1, from MPM on pleura. Therefore, these results suggest that intelectin-1 staining is an excellent histopathological tool for differentiating epithelioid-type MPMs from other cancers.

## Discussion

### Utility of Intelectin-1 Staining in Differential Diagnosis of MPM

It is difficult to distinguish MPM from metastatic tumors of other cancers. Pleura-invading lung adenocarcinoma cells, in particular, resemble epithelioid-type MPM cells in histopathology. Further, during immunohistological diagnosis, these cells are likely to be confused for each other because they often express the same antigens. CK5/6 and mesothelin are often expressed in lung and other cancers. Even calretinin, which is a typical MPM marker, was shown to be expressed in about 20% of lung adenocarcinomas (Table 2) [6]. In this study, we found that intelectin-1 is expressed by about 90% (23/26 cases) of epithelioid-type MPMs and by <5% (1/68 cases) of lung adenocarcinomas. Other cancers, except mucus-producing adenocarcinomas, were not stained significantly with anti-intelectin-1. The achromatic ratio of intelectin-1 in non-MPM tumors was better than that of other MPM markers. Mucus-producing metastatic cancer is rare in the pleura, and it is easy to discriminate between mucus-producing cancers and MPMs in the pleura. Thus, intelectin-1 staining would be useful in the differential diagnosis of epithelioid-type MPMs from other cancers.

Intelectin-1 staining is an excellent means of performing a differential diagnosis of MPM, not only due to its specificity for MPM but also due to the evidently higher positive proportion of MPMs (Table S1). The higher positive proportion could be useful for detection of MPM in a small biopsy-resected tissue. In addition, intelectin-1 staining is easy to observe in specimens because intelectin-1 expression is not detectable in most cells in normal tissues. Although podoplanin was a good marker of MPM with regard to specificity (Table 2 and 3), the staining of some stromal cells in tissues often interfered with podoplanin detection of MPMs (data not shown). Similarly, nuclear staining for WT-1 was specifically observed in MPMs (Tables 2 and 3) [5]; however, its use in differential diagnosis is likely to be limited because the antigen is often and strongly detected in the cytoplasm of various cancer cells and normal cells (data not shown). The simplicity of assessment using intelectin-1 staining ensures better differential diagnosis than when other mesothelioma markers are used. Therefore, intelectin-1 staining could be useful in diagnosing epithelioid-type MPMs, which account for 50–80% of MPMs, in cases suspected to be mesothelioma.

There were no non-MPM tumors, which were positive for both intelectin-1 and calretinin. In brief, epithelioid-type MPM could be distinguished from all tested cancers (n = 201) by double positive staining of intelectin-1 and calretinin at 88% of sensitivity and 100% of specificity. Non-epithelioid MPM expressed little intelectin-1, therefore intelectin-1 staining would be unable to replace all other MPM markers as a diagnostic marker for MPM. However, the complementary staining of intelectin-1 and calretinin could help diagnose epithelioid-type MPM. Furthermore, immunostaining of podoplanin and WT-1 would be useful for diagnosis of sarcomatoid-type MPM. The expression of these markers in metastatic tumor into the pleura is not well characterized. Further investigation is needed for designing a staining panel for pleural tumors using histological diagnostic markers, like cytokeratin and mucin antigen.

### Intelectin-1 Expression in Normal Tissues

Intelectin-1 was expressed mainly in the intestine. Mouse intelectin was reported to be expressed in intestinal paneth cells [9]. However, human intelectin-1 was pooled in mucous granules in goblet cells, but not in paneth cells. Although human intelectin-1/lactoferrin receptor has been reported to be a glycophosphatidylinositol-anchored membrane protein [19], intelectin-1 was obviously secreted along with mucus into the intestinal lumen (Figure 1, panel J) and was present as a soluble protein in mucus, adhering to microvilli rather than as a membrane-anchored protein. Human intelectin-1 could be a soluble protein secreted from intestinal goblet cells along with mucus.

Intelectin-1 was found to be expressed in the airway upon inflammation of the respiratory system [14]. In our study, intelectin-1 was occasionally detected in normal bronchial mucous gland, but not in goblet cells, ciliated cells, or serous cells in normal bronchus (Figure 2E and 2F). Inflammation of bronchi causes hyperplasia of goblet cells and an increase in the production of mucus containing MUC2 [20]. Intestinal goblet cells secrete MUC2 [21] and intelectin-1. Thus, the bronchial intelectin-1 may be expressed in MUC2-producing goblet cells in the bronchus in addition to the bronchial mucous gland.

Neither liver nor pancreas was stained with the monoclonal antibody against human intelectin-1. In a previous report, intelectin was detected in liver and pancreas, although polyclonal antibodies were used in the immunohistochemical studies [22]. Intelectin-1 mRNA has been detected in some organs besides the intestine, but not in liver and pancreas [10], [15]. Thus, the polyclonal antibodies against intelectin might bind to some antigens other than intelectin-1, like intelectin-2 [8], in these tissues.

Although intelectin-1 mRNA had been clearly detected in heart [10], intelectin-1 protein was expressed in only the mesothelial cells of the pericardium, but not the cardiac muscle or the endocardium. Similarly, intelectin-1/omentin was reported to be produced in adipocytes in omentum [16]; however, in this study, intelectin-1 was detected in mesothelial cells on adipose tissue rather than in adipocytes. The intelectin-1/HL-1 expression in vascular endothelial cells reported previously [15] was not confirmed in our results. Furthermore, intelectin-1 was not secreted from vascular endothelial cells cultured in vitro–human umbilical vein endothelial cells, unstimulated or stimulated with interleukin-4 or tumor necrosis factor-alpha (data not shown). These results suggested that the intelectin-1 expression detected in the heart or the omentum might have occurred in the mesothelial cells that covered the organs and that vascular endothelial cells express little intelectin-1 protein.

Occasional expression of intelectin-1 was observed in mesothelial cells, kidney collecting tubule cells, and umbrella cells in the bladder, suggesting that intelectin-1 was transiently expressed in these cells. The intelectin-1 in non-goblet cells, including MPMs, was diffusely detected in cytoplasm lacking large mucous granules. Since a viscous secretion with intelectin-1 was often observed in MPMs and malignant pleural effusions of MPMs contained intelectin-1 [8], the intelectin-1 in non-goblet cells could be secreted extracellularly, through the constitutive secretory pathway without accumulating into secretory vesicles first. The intelectin-1 production in non-goblet cells may be controlled by transcriptional regulation in a manner different from the one observed in goblet cells. Analysis of the transcriptional regulation of intelectin-1 in non-goblet cells may be helpful in identifying the reasons behind the specific expression of this antigen in MPMs.

## Supporting Information

Figure S1 **Specific binding of anti-intelectin-1 in mucus**. Specimens with goblet cells were immunostained with anti-intelectin-1 or a negative control antibody (anti-WT-1 monoclonal antibody (6F-H2)). The mucus was not stained with the antibody for the negative control.
(TIF)

Table S1 **Intensity and proportion of immunohistochemical staining of MPM against mesothelioma markers.** The intensity of staining was defined by applying the Allred scoring as follows: 3+, strong staining; 2+, moderate staining; 1+, weak staining; –, no staining. Representative photographs of staining are shown in Figure 3. The proportion of staining was measured for MPM cells in the entire microscopic field of each specimen. In the immunostaining of calretinin or WT-1, staining in the nucleus, but not the cytoplasm, was designated as a positive sample. CK5/6, cytokeratin 5, 6; MPM, malignant pleural mesothelioma; WT-1, Wilm’s tumor gene product 1.
(PDF)
