# Supplementary figures and images for: Specific Expression of Human Intelectin-1 in Malignant Pleural Mesothelioma and Gastrointestinal Goblet Cells
Source: PLoS One. 2012 Jul 2;7(7):e39889. doi: 10.1371/journal.pone.0039889 (PMC3388067; doi:10.1371/journal.pone.0039889)

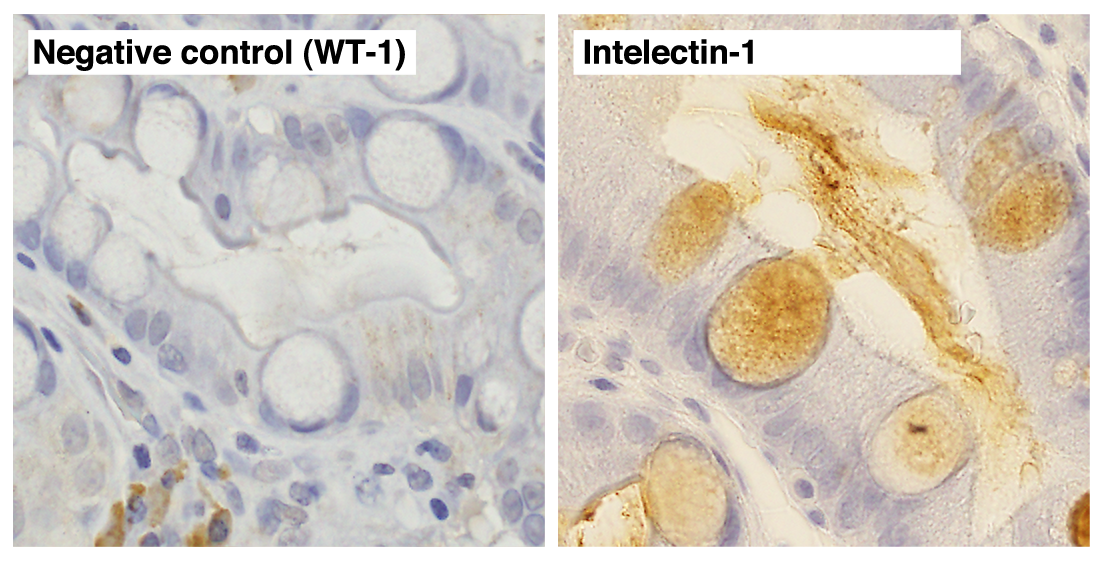

Supplement: Figure S1 — Specific binding of anti-intelectin-1 in mucus. Specimens with goblet cells were immunostained with anti-intelectin-1 or a negative control antibody (anti-WT-1 monoclonal antibody (6F-H2)). The mucus was not stained with the antibody for the negative control. (TIF) [file pone.0039889.s001.tif]
