# Supplementary material for: Specific Expression of Human Intelectin-1 in Malignant Pleural Mesothelioma and Gastrointestinal Goblet Cells
Source: PLoS One. 2012 Jul 2;7(7):e39889. doi: 10.1371/journal.pone.0039889 (PMC3388067; doi:10.1371/journal.pone.0039889)
Supplement: Table S1 — Intensity and proportion of immunohistochemical staining of MPM against mesothelioma markers. The intensity of staining was defined by applying the Allred scoring as follows: 3+, strong staining; 2+, moderate staining; 1+, weak staining; –, no staining. Representative photographs of staining are shown in Figure 3. The proportion of staining was measured for MPM cells in the entire microscopic field of each specimen. In the immunostaining of calretinin or WT-1, staining in the nucleus, but not the cytoplasm, was designated as a positive sample. CK5/6, cytokeratin 5, 6; MPM, malignant pleural mesothelioma; WT-1, Wilm’s tumor gene product 1. (PDF) [file pone.0039889.s002.pdf]

Table S1. Intensity and proportion of immunohistochemical staining of MPM against mesothelioma markers.

MPM type	ID	Intelectin-1	Calretinin	CK5/6	Podoplanin	WT-1	Mesothelin
Epithelioid	#1	3+ (90%)	2+ (80%)	2+ (90%)	2+ (80%)	3+ (90%)	2+ (90%)
	#2	2+ (99%)	2+ (30%)	2+ (80%)	2+ (40%)	2+ (80%)	2+ (10%)
	#3	2+ (99%)	2+ (80%)	1+ (30%)	-	2+ (60%)	2+ (40%)
	#4	2+ (70%)	2+ (50%)	3+ (90%)	1+ (5%)	2+ (40%)	2+ (70%)
	#5	1+ (5%)	2+ (70%)	1+ (1%)	2+ (80%)	2+ (70%)	2+ (30%)
	#6	2+ (80%)	2+ (70%)	2+ (90%)	2+ (20%)	2+ (30%)	2+ (20%)
	#7	2+ (90%)	2+ (70%)	2+ (50%)	2+ (40%)	1+ (5%)	2+ (70%)
	#8	2+ (60%)	2+ (30%)	3+ (90%)	2+ (30%)	2+ (40%)	2+ (30%)
	#9	2+ (50%)	2+ (50%)	3+ (99%)	2+ (20%)	2+ (80%)	2+ (10%)
	#10	-	2+ (30%)	2+ (90%)	2+ (20%)	2+ (50%)	2+ (90%)
	#11	2+ (80%)	2+ (80%)	2+ (50%)	2+ (80%)	2+ (70%)	2+ (60%)
	#12	2+ (1%)	2+ (30%)	2+ (70%)	-	2+ (30%)	2+ (10%)
	#13	2+ (5%)	2+ (30%)	2+ (30%)	2+ (40%)	2+ (80%)	2+ (50%)
	#14	2+ (30%)	2+ (80%)	2+ (90%)	2+ (90%)	2+ (80%)	3+ (99%)
	#15	2+ (40%)	2+ (10%)	2+ (70%)	2+ (70%)	2+ (80%)	2+ (10%)
	#16	2+ (99%)	2+ (90%)	2+ (99%)	2+ (70%)	2+ (90%)	2+ (70%)
	#17	2+ (90%)	2+ (90%)	2+ (30%)	2+ (80%)	-	2+ (60%)
	#18	2+ (99%)	2+ (10%)	2+ (99%)	2+ (90%)	-	2+ (80%)
	#19	2+ (99%)	2+ (80%)	2+ (80%)	1+ (80%)	2+ (60%)	2+ (50%)
	#20	3+ (95%)	2+ (50%)	2+ (90%)	2+ (30%)	2+ (70%)	1+ (5%)
	#21	2+ (30%)	2+ (30%)	2+ (70%)	2+ (60%)	2+ (80%)	1+ (20%)
	#22	3+ (99%)	3+ (99%)	2+ (90%)	2+ (60%)	2+ (80%)	2+ (80%)
	#23	3+ (99%)	2+ (80%)	2+ (90%)	2+ (80%)	2+ (20%)	2+ (90%)
	#24	2+ (99%)	1+ (20%)	2+ (40%)	2+ (40%)	2+ (99%)	1+ (20%)
	#25	-	2+ (40%)	2+ (10%)	2+ (50%)	2+ (70%)	2+ (40%)
	#26	2+ (5%)	2+ (50%)	-	2+ (10%)	2+ (80%)	2+ (30%)
Poorly differentiated	#27	-	-	2+ (80%)	1+ (10%)	2+ (50%)	2+ (50%)
	#28	-	-	2+ (20%)	2+ (80%)	1+ (10%)	-
	#29	2+ (40%)	2+ (50%)	2+ (70%)	2+ (30%)	2+ (50%)	1+ (20%)
Biphasic	#30	-	2+ (30%)	2+ (5%)	2+ (50%)	1+ (5%)	-
	#31	1+ (1%)	2+ (70%)	2+ (80%)	2+ (70%)	2+ (50%)	2+ (50%)
	#32	2+ (10%)	2+ (30%)	2+ (50%)	-	2+ (30%)	1+ (10%)
Sarcomatoid	#33	-	-	-	2+ (20%)	2+ (30%)	-
	#34	-	1+ (5%)	1+ (10%)	2+ (80%)	2+ (40%)	-
	#35	-	1+ (5%)	2+ (5%)	1+ (5%)	2+ (20%)	-
	#36	1+ (<1%)	1+ (5%)	1+ (1%)	1+ (30%)	2+ (1%)	-
	#37	-	-	-	2+ (90%)	2+ (10%)	-
	#38	1+ (5%)	-	2+ (1%)	-	-	-
	#39	-	-	-	-	-	-

The intensity of staining was defined by applying the Allred scoring as follows: 3+, strong staining; 2+, moderate staining; 1+, weak staining; –, no staining. Representative photographs of staining are shown in Figure 3. The proportion of staining was measured for MPM cells in the entire microscopic field of each specimen. In the immunostaining of calretinin or WT-1, staining in the nucleus, but not the cytoplasm, was designated as a positive sample. CK5/6, cytokeratin 5, 6; MPM, malignant pleural mesothelioma; WT-1, Wilm’s tumor gene product 1.
